# Prevalence and risk factors of oral human papillomavirus infection among 4212 healthy adults in Hebei, China

**DOI:** 10.1186/s12879-023-08759-y

**Published:** 2023-11-08

**Authors:** Shuting Yu, Yingying Zhu, Huijing He, Yaoda Hu, Xiaoli Zhu, Wenwen Diao, Shuguang Li, Guangliang Shan, Xingming Chen

**Affiliations:** 1 Department of Otolaryngology-Head and Neck Surgery, Peking Union Medical College Hospital, Peking Union Medical College and Chinese Academy of Medical Sciences, No. 1 Shuaifuyuan, Dongcheng District, Beijing, China; 2 Department of Epidemiology and Statistics, Institute of Basic Medical Sciences, Chinese Academy of Medical Sciences & School of Basic Medicine, Peking Union Medical College, 5 Dongdansantiao, Dongcheng District, Beijing, 100005 China; 3 Department of Music Artificial Intelligence and Music Information Technology, Central Conservatory of Music, 43 Baojiajie, Xicheng District, Beijing, China

**Keywords:** Human papillomavirus, Oral infection, Risk factors, China

## Abstract

**Background:**

Human papillomavirus (HPV) infection is an essential cause of oropharyngeal squamous cell carcinoma that is increasing in incidence worldwide. However, little is known about the epidemiology of oral HPV infection among healthy adults in China.

**Methods:**

A study in northern China was conducted in 2021 as baseline data of Diverse Life-Course Cohort (DLCC). Residents who aged above 20 were eligible to participate. Oral swab specimens and questionnaires were collected from 4226 participants. HPV DNA in oral exfoliated cells was tested by Nested Polymerase Chain Reaction approach and sequencing. Univariate and multivariate analyses were performed to assess the associations between exposure factors and oral HPV infection.

**Results:**

Overall prevalence of oral HPV infection was 4.08% (95%CI, 3.69%-4.68%). The most prevalent HPV type detected was HPV-81 (1.35%; 95% CI, 1.00%–1.70%), followed by HPV-16 (0.64%; 95% CI, 0.40%–0.88%). Oral HPV infection presented a bimodal pattern with respect to age in male and female participants. Oral HPV prevalence of male participants was significantly higher than prevalence of female participants (5.0% versus 3.6%, *P* = 0.041). Prevalence of oral HPV was higher among current smokers (OR = 1.59; 95% CI, 1.11–2.29; *P* = 0.039) and current drinkers (OR = 1.60; 95% CI, 1.14–2.25; *P* = 0.023). Current alcohol consumption was independently associated with oral HPV infection (OR = 1.74; 95% CI, 1.22–2.50; *P* = 0.010).

**Conclusions:**

Among healthy adults aged above 20 in Hebei, China, the prevalence of high-risk HPV infection was 1.92% (95%CI, 1.51%-2.34%). Oral HPV prevalence was independently associated with alcohol consumption. More tailored prevention strategies are needed to prevent oral HPV infection through smoking cessation, reduction of alcohol consumption, and HPV vaccination.

**Supplementary Information:**

The online version contains supplementary material available at 10.1186/s12879-023-08759-y.

## Introduction

As a common sexually transmitted disease in many countries, the oncogenic character of human papillomavirus (HPV) was well studied in cervical cancer. Forty years ago, the link between HPV infection and head and neck carcinoma was uncovered by Gissmann et al. [1]. Since then, more and more studies identified the important role of high-risk HPV infection in the etiology of oropharyngeal squamous cell carcinoma (OPSCC) [2, 3]. It is estimated that about 70% of OSPCC cases are related to oral HPV infection in North America [4, 5].

Due to decreasing alcohol and tobacco consumption over the last 3 decades, the incidence of HPV-negative OSPCC declined by 50% in developed countries [4]. However, incidence of overall OSPCC increased rapidly in recent years, not only in western countries, but also in China, mainly owing to the rise in HPV-related OSPCC [4, 6–8]. HPV-positive OSPCC patients in western countries tend to be male, young, and white individuals [4]. Besides, these patients have distinct clinical features and favorable prognosis [9]. Therefore, it is significant to understand the prevalence and risk factors of oral HPV infection in healthy population.

The reported prevalence of oral HPV infection in healthy population ranged from 2 to 15% [10–12]. The variation may reflect differences in study population, sample size, specimen collection, and detection methodology. Up to now, large studies of oral HPV infection were mainly conducted in the United States. Although China has a vast territory and a large population, there are few studies exploring the epidemiology of oral HPV infection in healthy Chinese individuals. This baseline study aimed to investigate the prevalence and risk factors of oral HPV infection in a large Chinese population from urban and rural areas.

## Methods

### Study population

The present study was derived from the Diverse Life-Course Cohort (DLCC) [13], which is a population-based prospective cohort study conducted by the Institute of Basic Medical Sciences (IBMS), Chinese Academy of Medical Sciences (CAMS). This is a cross-sectional study conducted in 2021, designing to investigate oral HPV infection in 2 areas from Hebei province (Baoding and Laiyuan) in mainland China. Residents who aged above 20 and lived in the local area for at least 1 year were eligible to participate. Pregnant women, soldiers in service, patients with severe mental diseases, and disabled people were excluded.

The study has been conducted in accordance with the Declaration of Helsinki. The study was approved by the Bioethical Committee of the Institute of Basic Medical Sciences, Chinese Academy of Medical Sciences (No. 055–2020). A signed informed consent was obtained from each participant before the survey.

### Demographic and behavioral data

Self-reported questionnaires were applied to collect the demographic and behavioral data as previously described [14], including education level, income level, marital status, type of employment, exercise, HPV vaccination, tobacco and alcohol consumption. Current smokers were defined as people who smoked at least one cigarette per day and lasted for at least 6 months. Current drinkers were defined as people who consumed at least 50 g alcohol or 1 bottle of beer for at least twice a month. Physical activity at work was divided into three categories as light, moderate, or heavy according to intensity, as previously described [15]. Moderate or vigorous activity for at least 20 min was delimited as exercise. Chronic pharyngitis was diagnosed if a participant complained discomfort, pain, tickling sensation in the throat and displayed corresponding signs in oral examination. Trained interviewers and qualified program managers improved the validity of the self-reported data [14]. Oropharyngeal examination was accomplished by an otolaryngologist.

### Oral sample collection

Following oropharyngeal examination, a trained otolaryngologist used a sterile oral swab from the Virus Specimen Collection Kit (Guangzhou Darui Biotechnology Co., Ltd.) to collect epithelial cells by rubbing 5 times at each site: the buccal mucosa, palate, tonsils, top and bottom of the tongue, the inner upper and lower lips, the gingival surfaces. These swabs were stored in preservation solution from the same Kit (Guangzhou Darui Biotechnology Co., Ltd.). Samples were frozen at -80 °C and shipped for further test.

### DNA purification, HPV detection and genotyping

DNA extraction was carried out on a NAE-96/24 automated workstation using the E.Z.N.A. Mag-Bind Tissue DNA Kit (M6223; Omega Bio-Tek, Inc.). DNA quality was assessed by the amplification of the β-globin gene using the PC03 and PC04 primers. HPV DNA in the β-globin positive samples was tested using a highly sensitive and specific Nested Polymerase Chain Reaction (Nest PCR) approach consisting of the MY09/11 primer set (primary PCR) and the GP5 + /6 + primer set [16]. HPV types were determined by cloning and sequencing. The obtained results were subsequently analyzed with BLAST database (http://blast.ncbi.nlm.nih.gov). In this way, a broad spectrum of HPV types could be tested (HPV 3, 6, 7, 10, 11, 16, 18, 26, 27, 29, 30, 31, 32, 33, 35, 37, 39, 40, 42, 43, 44, 45, 51, 52, 53, 54, 55, 56, 57, 58, 59, 61, 62, 66, 67, 68, 69, 70, 72, 74, 75, 77, 81, 82, 84, 87, 90, 91, and 94) [17]. HPV types are defined as high-risk or low-risk based on epidemiological associations with cervical cancer [18]. High-risk HPV types include HPV 16, 18, 26, 31, 33, 35, 39, 45, 51, 52, 53, 56, 58, 59, 66, 68, 73, 82. Low-risk HPV types include HPV 6, 11, 40, 42, 54, 55, 61, 62, 64, 67, 69, 70, 71, 72, 81, 82, 83, 84, 89. Rigorous quality control procedures were conducted to avoid false-positive and false-negative results, as described [19, 20]. The following negative and positive controls were included in each 96-well PCR reaction plate: 4 controls without any DNA template; 2 sets of CaSki cells genome DNA (HPV 16 positive), each containing 1 ng and 0.1 ng genome DNA (through calculating, 1 ng genome DNA represents approximately 60,000 viral copies [21–24]. Test results were adopted only when controls met the following criteria: (i) all 4 negative controls were negative and (ii) at least one set of the CaSki cells genome DNA controls were positive and the signal intensity matched input copy number.

### Statistical analysis

Baseline characteristics of participants were presented as numbers and percentages. HPV prevalence was reported as percentage and 95% confidence intervals (95%CI) calculated with binomial distribution. Associations with oral HPV prevalence were calculated as unadjusted and adjusted odds ratios (OR) and 95% CI using binomial logistic regression models. Two-sided *P* values < 0.05 were considered statistically significant. Prevalence at each age point was weighted and plotted with weighted smoothing algorithm by Python [25].

## Results

### Basic characteristics

Investigation of HPV prevalence included 4226 participants. All samples were β-globin positive. Among all participants, 4212 (99.69%) finished all self-reported questionnaires. Baseline characteristics of these participants were shown in Table 1 and Supplementary Table 1. The median age of participants was 58. As presented in Table 1, 75.3% of respondents were over 50 years old. The female: male ratio was 2.03:1. Eighty-five percent of participants had education level of less than college, and 87.16% of participants were married. The majority of people engaged in light physical activity at work (75.66%). Seventy percent of participants exercised at least one time per week. Male constituted 95.91% of current smokers and 85.03% of current drinkers. Only 1.38% of females received HPV vaccination. A small percentage of subjects (8.00%) showed chronic pharyngitis at physical examination.

**Table 1:** Baseline characteristics of the participants

	Male		Female		Overall		Any oral HPV infection	
Characteristics	No	%	No	%	No	%	No	%
Age (years)								
20–29	42	3.03%	85	3.01%	127	3.02%	4	3.1%
30–39	107	7.71%	226	8.00%	333	7.91%	12	3.6%
40–49	187	13.47%	392	13.88%	579	13.75%	22	3.8%
50–59	339	24.42%	931	32.97%	1270	30.15%	56	4.4%
60–69	477	34.37%	805	28.51%	1282	30.44%	47	3.7%
≥ 70	236	17.00%	385	13.63%	621	14.74%	31	5.0%
Sex								
Female			2824		2824	67.05%	103	3.6%
Male	1388				1388	32.95%	69	5.0%
Area								
Urban area: Baoding	543	39.12%	1308	46.32%	1851	43.95%	78	4.2%
Rural area: Laiyuan	845	60.88%	1516	53.68%	2361	56.05%	94	4.0%
Marital status								
Never married	59	4.25%	56	1.98%	115	2.73%	3	2.6%
Married	1249	89.99%	2422	85.76%	3671	87.16%	150	4.1%
Widowed/divorced/separated	80	5.76%	342	12.11%	422	10.02%	18	4.3%
Missing	0	0.00%	4	0.14%	4	0.09%	1	25.0%
Cigarette use								
Never	420	30.26%	2788	98.73%	3208	76.16%	118	3.7%
Former	265	19.09%	6	0.21%	271	6.43%	12	4.4%
Current	703	50.65%	30	1.06%	733	17.40%	42	5.7%
Alcohol consumption								
Never	470	33.86%	2684	95.04%	3154	74.88%	115	3.6%
Former	174	12.54%	9	0.32%	183	4.34%	7	3.8%
Current	744	53.60%	131	4.64%	875	20.77%	50	5.7%
HPV vaccination								
Never	1113	80.19%	2756	97.59%	3869	91.86%	160	4.1%
Ever	0	0.00%	39	1.38%	39	0.93%	1	2.6%
Missing	275	19.81%	29	1.03%	304	7.22%	11	3.6%
Education								
Illiterate or elementary school	383	27.59%	991	35.09%	1374	32.62%	60	4.4%
Junior or senior high school	758	54.61%	1447	51.24%	2205	52.35%	83	3.8%
College or above	244	17.58%	384	13.60%	628	14.91%	29	4.6%
missing	3	0.22%	2	0.07%	5	0.12%	0	0.0%

### HPV prevalence and type distribution

**Fig. 1:**
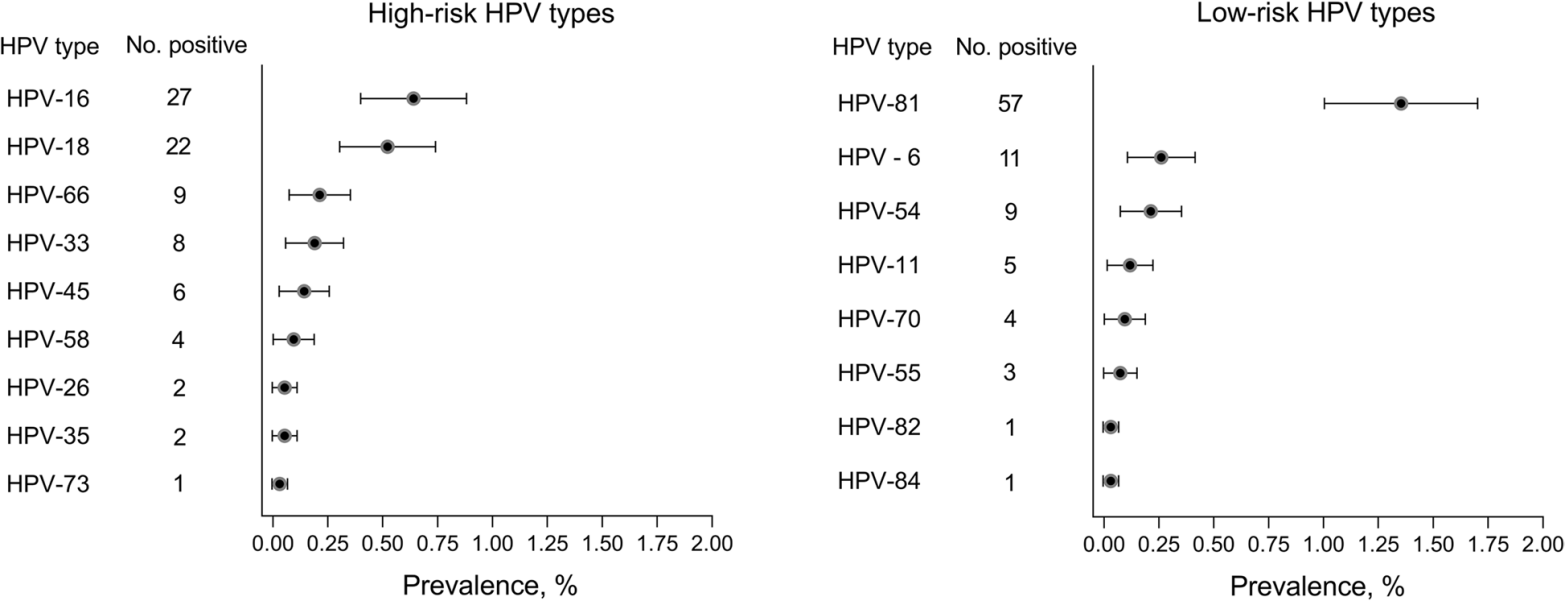
Type-specific prevalence of oral HPV infection among individuals aged above 20 years from Hebei, China

Of the 4212 samples, overall prevalence of oral HPV infection was 4.08% (95%CI, 3.69%-4.68%). The prevalence of high-risk HPV infection was 1.92% (95%CI, 1.51%-2.34%), and for low-risk HPV infections was 2.16% (95% CI, 1.72%–2.60%). The type-specific prevalence for the 17 HPV types was shown in Fig. 1. The most prevalent HPV type was HPV-81 (1.35%; 95% CI, 1.00%–1.70%), followed by HPV-16 (0.64%; 95% CI, 0.40%–0.88%) and HPV-18 (0.52%; 95% CI, 0.30%–0.74%). Multiple infections were uncommon, with only two participants positive for dual types (HPV 18 and 16; HPV 45 and 16).

### Factors associated with oral HPV infection

Logistic regression models were applied to analyze factors associated with prevalent oral HPV infection, which was shown in Table 2 and Supplementary Table 2. In univariate analysis, the prevalence of oral HPV tended to increase with age and was the highest in subjects older than 70 years of age (Table 1, 5.0%), although no significant difference in the prevalence of oral HPV was observed across age groups. Men had significantly higher prevalence of oral HPV than women (5% versus 3.6%; *P* = 0.041). Oral HPV prevalence of participants in urban area (Baoding) was similar to the prevalence of participants in rural area (Laiyuan) (4.2% versus 4.0%, *P* = 0.705). Participants with varying education level and income level didn’t show significant difference in oral HPV prevalence. Current smokers showed significantly higher prevalence of oral HPV then never smokers (Table 2, OR = 1.59; 95% CI, 1.11–2.29; *P* = 0.039). The oral HPV prevalence of current drinkers was also significantly higher than that of never drinkers (Table 2, OR = 1.60; 95% CI, 1.14–2.25; *P* = 0.023). Participants with vaccination presented lower oral HPV prevalence than the others (2.6% versus 4.1%). As only 39 out of 4212 participants received HPV vaccination, no statistical difference was observed. There was no significant association of oral HPV infection with physical activity at work, marital status, oropharyngeal examination, or exercise. In multivariate analysis including all the variables in Table 1 and Supplementary Table 1, only current alcohol consumption was independently associated with HPV infection (OR = 1.74; 95% CI, 1.22–2.50; *P* = 0.010). As some factors were unevenly distributed among males and females, we divided participants into two groups by gender and analyzed associations of these factors with oral HPV infection in the two subgroups. Results were shown in Supplementary Table 3.

**Table 2:** Factors associated with oral HPV infection in univariate and multivariate analyses

	Any oral HPV infection	
Characteristics	unadjusted OR (95% CI)	adjusted OR (95% CI)
Age (years)		
20–29	1.00	1.00
30–39	1.15(0.36–3.63)	1.06(0.33–3.42)
40–49	1.22(0.41–3.59)	1.30(0.43–3.97)
50–59	1.42(0.51–3.98)	1.58(0.54–4.65)
60–69	1.17(0.42–3.30)	1.07(0.36–3.20)
≥ 70	1.62(0.56–4.66)	1.34(0.43–4.15)
*P* value	0.728	0.543
Sex		
Male	1.00	1.00
Female	0.72(0.53–0.99)	0.79(0.51–1.22)
*P* value	0.041	0.289
Area		
Urban area: Baoding	1.00	1.00
Rural area: Laiyuan	0.94(0.69–1.28)	0.93(0.63–1.37)
*P* value	0.705	0.710
Marital status		
Never married	1.00	1.00
Married	1.59(0.50–5.06)	1.44(0.36–5.67)
Widowed/divorced/separated	1.66(0.48–5.75)	1.49(0.34–6.48)
Missing		
*P* value	0.714	0.868
Cigarette use		
Never	1.00	1.00
Former	1.21(0.66–2.23)	1.095(0.49–2.46)
Current	1.59(1.11–2.29)	1.22(0.66–2.25)
*P* value	0.039	0.812
Alcohol consumption		
Never	1.00	1.00
Former	1.05(0.48–2.29)	0.98(0.39–2.44)
Current	1.60(1.14–2.25)	1.74(1.22–2.50)
*P* value	0.023	0.010
HPV vaccination		
Never	1.00	1.00
Ever	0.61(0.08–4.47)	0.73(0.10–5.53)
Missing		
*P* value	0.623	0.757
Education		
Illiterate or elementary school	1.00	1.00
Junior or senior high school	0.86(0.61–1.20)	0.81(0.57–1.63)
College or above	1.06(0.67–1.67)	0.99(0.62–1.60)
missing		
*P* value	0.521	0.458

**Fig. 2:**
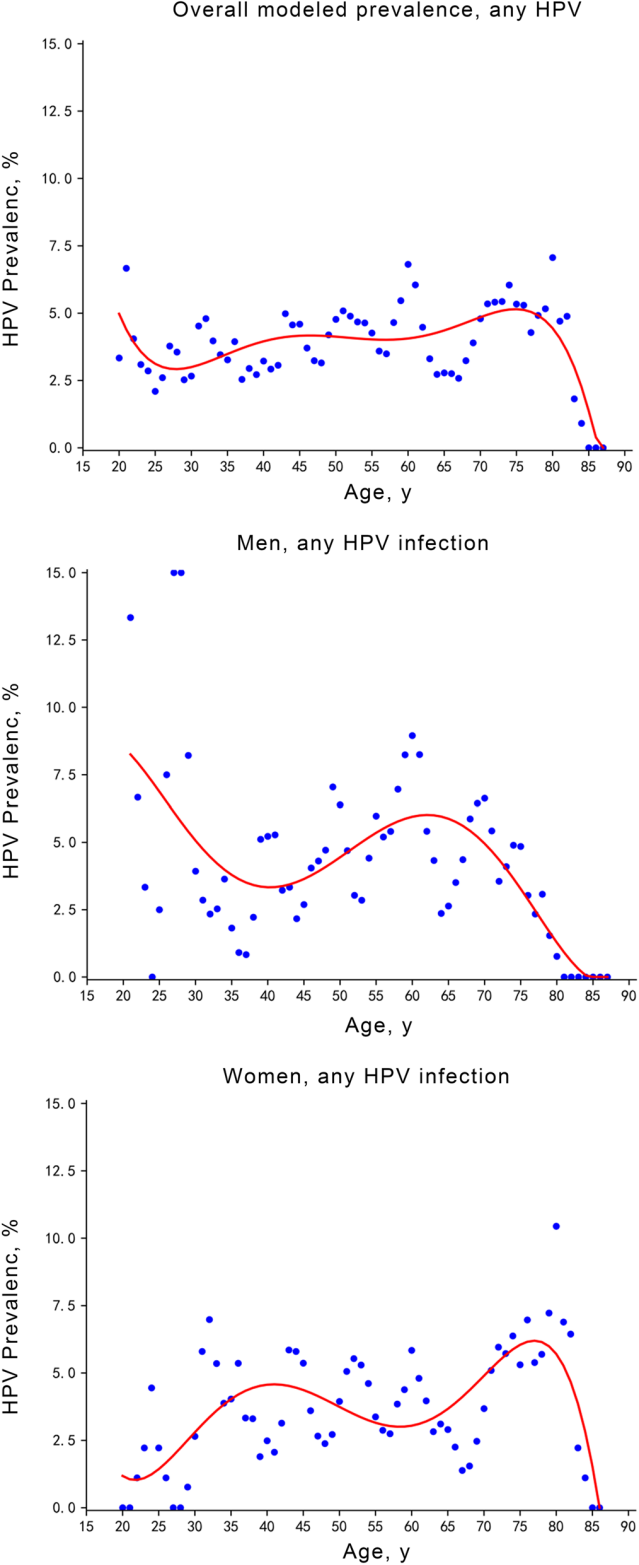
Modeled HPV prevalence across age among 4212 individuals aged above 20 years from Hebei, China

HPV prevalence across age was plotted and modeled in all participants, men, and women, respectively. As shown in Fig. 2, HPV prevalence across age in men and women presented a bimodal pattern. In male participants, the first peak was observed among those aged around 20 years, with the second peak among those aged around 63 years. In female participants, however, the first peak in prevalence was among those aged around 40 years and the second, higher peak was among those aged around 76 years.

## Discussion

Since the relationship between oral HPV infection and OPSCC had been uncovered, plenty of studies investigated the prevalence of oral HPV in healthy participants. Wide range of HPV prevalence was reported in literature, from highest prevalence in South America (12.4%; 95% CI = 5.7–19.1%) to lowest prevalence in Asia (2.6%; 95% CI = 0.6–4.6%) [11]. Besides differences in specimen collection and detection procedures, the reason for the variation in overall prevalence probably owes to diversity in the geographic distribution, sample size, and population risk level of the included studies. However, only a few large population-based studies have been conducted, mainly in the United States and Europe. Oral HPV infection in mainland China is underestimated due to lack of similar studies.

The first population-based investigation of oral HPV infection was conducted during 2009–2010, including 5579 healthy participants in the United States. The prevalence of oral HPV infection aged 14 to 69 years was 6.9% (95% CI, 5.7%–8.3%) [26], in which the majority were high-risk HPV infections (3.7%; 95% CI, 3.0%–4.6%). At the same time, a pioneer population-based study by Yang Ke and colleagues in China during 2009–2011 reported oral mucosal HPV prevalence of 0.67% (95% CI, 0.47%-0.93%), which was much lower than the above study [27]. Besides, only 1.1% of Chinese participants in the study declared more than one sexual partner, compared to 73.7% in the U.S., and only 3.94% of participants reported oral sexual practice, compared to 83.1% in the States. These huge differences in sexual activity might explain the distinct HPV prevalence of two countries.

In 2018, a Meta-analysis including 66 studies with total sample size of 56600 reported an overall HPV prevalence of 7.7%, in which only 3 studies were conducted in Asia [11]. Forty studies included in subgroup-analysis gave an overall high-risk HPV prevalence of 3.5% (95% CI, 2.5–4.7%). In the present study, overall oral HPV prevalence was 4.08% (95%CI, 3.69%-4.68%), which was different from other large-scale studies in China [12, 27], probably owes to diversity in the geographic distribution, sample size, specimen collection, detection technology, and population risk level. It is worth noting that high-risk HPV infection constituted nearly half of oral HPV infection (1.92%) in this study, which was much higher than previous investigations based on Chinese population [12, 27].

In large-scale investigations, reported cervical HPV prevalence in Chinese population ranged from 16.95% to 26.92% [28–30], which was much higher than prevalence of female oral HPV in this study. It was hypothesized that an immune response to HPV through sexual intercourse is much stronger than an immune response elicited from oral sex [31]. Therefore, genital HPV infection could theoretically confer protection against subsequent oral infection [32], which might partly explain the lower oral HPV prevalence.

In the present study, oral HPV infection presented a bimodal distribution with age in women, with higher peak among individuals aged 75 to 80 years. Bimodal distribution was also observed in a large-scale study [33], with a younger age of the second peak. The first peak in oral HPV prevalence might due to active sexual behavior, which needs further exploration. The second peak in oral HPV prevalence could be explained by reactivation of latent infections because of age-related loss of immunity [34], increased persistence among older participants [35], or differences in sexual behaviors across ages [36]. Besides, given the lack of screening and treatment for oral HPV infections, the cumulative prevalence of chronic infections might increase with age.

According to literature, there has been an upward trend in the prevalence of oral and oropharyngeal cancer in the elderly. In the United States, rates for all cancers of the oral cavity and pharynx combined increased among persons aged 50–79 years over recent years [37]. In China, the age-specific incidence rate dramatically increased after 40 years and peaked in the age group of 80 − 84 years for both sexes [38]. In present study, there was an evident peak of oral HPV infection in both genders in elder participants, corresponding to previous studies [26], which revealed a possible cause of age distribution of oral and oropharyngeal cancer. Therefore, the age-specific prevalence of oral HPV infection needs further examination in large-scale surveys, and more attention should be devoted to the prevention of oral HPV infection in the elderly.

The prevalence of overall oral HPV infection among male participants was significantly higher than oral HPV infection among female participants in this study, which is consistent with most studies in western countries [11]. Thirty-three percent of participants in this study were male, and only 3% male participants were under 30. Since young male participants showed highest oral HPV prevalence, it is likely that oral HPV infection was still underestimated in men.

Cigarette and alcohol consumption were common among men (50.65% and 53.60%) in this study. Our data indicated that smoking was positively associated with oral HPV infection, which had been proven in other studies [12, 26]. Immunosuppressive effects of smoking might be the underlying cause [39]. In this study, alcohol consumption was independently associated with oral HPV prevalence, corresponding with previous study [12, 26], which could due to the systemic effects of alcohol on immune function [40, 41]. Further longitudinal analyses will be needed to explore whether alcohol consumption is related with oral HPV acquisition and clearance.

HPV vaccination rate of women was only 1.4% in our investigation, which is much lower than that reported in western countries [33]. Besides lack of vaccine availability, vaccine cost and insufficient knowledge of HPV might also cause the low HPV vaccination rate in China [42]. In addition to the low vaccination rate among females, it is also worth noting that vaccination is not yet approved for males in China, therefore, little was known on effectiveness of HPV vaccine in males. HPV vaccine had already shown herd protection against cervical as well as oral HPV infections [33], therefore, it is urgent to boost HPV vaccination rate in China.

There were several limitations in this study. The investigation was community-based and mostly conducted in suburbs, where young people tended to go to larger cities for work. Thus, over 75% of the participants were over 50 and held a conservative attitude about sex, which increased the difficulty in collecting sexual behaviors. Limited education level of participants also contributed to the loss of sexual behavioral data. Future study with more rigorous design will be conducted to collect sexual behavior including oral sex practices. Uneven distribution of gender and age in participants might lead to biased estimation of oral HPV infection. Given the low prevalence of oral HPV, statistical power might be insufficient to detect the associations of confounding factors. In view of the cross-sectional nature of this study, observed associations cannot be interpreted as temporally linked with infection, and the accurate reasons for a bimodal age pattern are mostly unknown and challenging to detect. Large-scale studies including other geographic locations and prospective natural history studies are needed to further explore epidemiology and risk factors of oral HPV infection in China.

### Supplementary Information

**Additional file 1:** **Supplementary Table 1.** Baseline characteristics of the participants (not shown in article). **Supplementary Table 2.** Factors associated with oral HPV infection in univariate and multivariate analyses (not shown in article). **Supplementary Table 3.** Factors associated with oral HPV infection in males and females.

## Data Availability

The data is available at generalist data repository, https://doi.org/10.6084/m9.figshare.22300192.
