# Supplementary material for: Prevalence and risk factors of oral human papillomavirus infection among 4212 healthy adults in Hebei, China
Source: BMC Infect Dis. 2023 Nov 8;23:773. doi: 10.1186/s12879-023-08759-y (PMC10634102; doi:10.1186/s12879-023-08759-y)
Supplement: Supplementary file 1 — Additional file 1: Supplementary Table 1. Baseline characteristics of the participants (not shown in article). Supplementary Table 2. Factors associated with oral HPV infection in univariate and multivariate analyses (not shown in article). Supplementary Table 3. Factors associated with oral HPV infection in males and females. [file 12879_2023_8759_MOESM1_ESM.docx]

**Supplementary Table 1** Baseline characteristics of the participants (not shown in article)

|  | Male | |  | Female | |  | Overall | |  | Any oral HPV infection | |
| --- | --- | --- | --- | --- | --- | --- | --- | --- | --- | --- | --- |
| Characteristics | No. | % |  | No. | % |  | No. | % |  | No. | % |
| Income-to-poverty ratio |  |  |  |  |  |  |  |  |  |  |  |
| ≤1 | 293 | 21.11% |  | 687 | 24.33% |  | 980 | 23.27% |  | 38 | 3.9% |
| >1.0 to≤5.0 | 286 | 20.61% |  | 770 | 27.27% |  | 1056 | 25.07% |  | 42 | 4.0% |
| >5.0 to≤10.0 | 289 | 20.82% |  | 924 | 32.72% |  | 1213 | 28.80% |  | 52 | 4.3% |
| >5.0 to≤20.0 | 430 | 30.98% |  | 379 | 13.42% |  | 809 | 19.21% |  | 35 | 4.3% |
| ≥20.0 | 74 | 5.33% |  | 36 | 1.27% |  | 110 | 2.61% |  | 4 | 3.6% |
| Missing | 16 | 1.15% |  | 28 | 0.99% |  | 44 | 1.04% |  | 1 | 2.3% |
| P value | 0.979 |  |  |  |  |  |  |  |  |  |  |
| Physical activity at work |  |  |  |  |  |  |  |  |  |  |  |
| Light | 825 | 59.44% |  | 2362 | 83.64% |  | 3187 | 75.66% |  | 134 | 4.2% |
| Moderate | 224 | 16.14% |  | 291 | 10.30% |  | 515 | 12.23% |  | 17 | 3.3% |
| Heavy | 339 | 24.42% |  | 171 | 6.06% |  | 510 | 12.11% |  | 21 | 4.1% |
| P value | 0.629 |  |  |  |  |  |  |  |  |  |  |
| Oropharyngeal examination |  |  |  |  |  |  |  |  |  |  |  |
| Normal | 1231 | 88.69% |  | 2520 | 89.24% |  | 3751 | 89.06% |  | 151 | 4.0% |
| Chronic pharyngitis | 120 | 8.65% |  | 217 | 7.68% |  | 337 | 8.00% |  | 16 | 4.7% |
| Tonsillar Hypertrophy | 10 | 0.72% |  | 48 | 1.70% |  | 58 | 1.38% |  | 0 | 0.0% |
| Others | 27 | 1.95% |  | 39 | 1.38% |  | 66 | 1.57% |  | 5 | 7.6% |
| P value | 0.177 |  |  |  |  |  |  |  |  |  |  |
| Exercise |  |  |  |  |  |  |  |  |  |  |  |
| 5-7 days/week | 727 | 52.38% |  | 1498 | 53.05% |  | 2225 | 52.83% |  | 91 | 4.1% |
| 1-4 days/week | 216 | 15.56% |  | 528 | 18.70% |  | 744 | 17.66% |  | 28 | 3.8% |
| ≤3days/month | 445 | 32.06% |  | 798 | 28.26% |  | 1243 | 29.51% |  | 53 | 4.3% |
| P value | 0.862 |  |  |  |  |  |  |  |  |  |  |

**Supplementary Table 2** Factors associated with oral HPV infection in univariate and multivariate analyses (not shown in article)

|  | Any oral HPV infection | | |
| --- | --- | --- | --- |
| Characteristics | unadjusted OR (95% CI) |  | adjusted OR (95% CI) |
| Income-to-poverty ratio |  |  |  |
| ≤1 | 1.00 |  | 1.00 |
| >1.0 to≤5.0 | 1.03(0.66-1.61) |  | 1.14(0.69-1.88) |
| >5.0 to≤10.0 | 1.11(0.72-1.70) |  | 1.13(0.64-2.01) |
| >5.0 to≤20.0 | 1.12(0.70-1.79) |  | 0.97(0.51-1.99) |
| ≥20.0 | 0.94(0.33-2.67) |  | 0.81(0.24-2.69) |
| Missing |  |  |  |
| P value | 0.979 |  | 0.928 |
| Physical activity at work |  |  |  |
| Light | 1.00 |  | 1.00 |
| Moderate | 0.78(0.47-1.30) |  | 0.72(0.42-1.25) |
| Heavy | 0.98(0.61-1.57) |  | 0.81(0.48-1.40) |
| P value | 0.629 |  | 0.437 |
| Oropharyngeal examination |  |  |  |
| Normal | 1.00 |  | 1.00 |
| Chronic pharyngitis | 1.19(0.71-2.01) |  | 0.82(0.48-1.42) |
| Tonsillar Hypertrophy | 0.00 |  | 0.00 |
| Others | 1.95(0.77-4.93) |  | 1.70(0.59-4.89) |
| P value | 0.177 |  | 0.434 |
| Exercise |  |  |  |
| 5-7 days/week | 1.00 |  | 1.00 |
| 1-4 days/week | 0.92(0.60-1.41) |  | 0.95(0.60-1.51) |
| ≤3days/month | 1.04(0.74-1.48) |  | 1.08(0.73-1.60) |
| P value | 0.862 |  | 0.881 |

**Supplementary Table 3** Factors associated with oral HPV infection in males and females

|  | unadjusted OR (95% CI) | | |
| --- | --- | --- | --- |
| Characteristics | Male |  | Female |
| Marital status |  |  |  |
| Never married | 1.00 |  | 1.00 |
| Married | 1.49(0.36-6.24) |  | 2.07(0.28-15.16) |
| Widowed/divorced/separated | 1.90(0.36-10.15) |  | 2.17(0.28-16.95) |
| Missing |  |  |  |
| P value | 0.745 |  | 0.751 |
| Cigarette use |  |  |  |
| Never | 1.00 |  | 1.00 |
| Former | 0.83(0.38-1.81) |  | 13.59(2.46-75.03) |
| Current | 1.27(0.73-2.23) |  | 1.94(0.46-8.26) |
| P value | 0.417 |  | 0.008 |
| Alcohol consumption |  |  |  |
| Never | 1.00 |  | 1.00 |
| Former | 0.90(0.37-2.15) |  | 0 |
| Current | 1.25(0.73-2.14) |  | 2.03(1.00-4.12) |
| P value | 0.594 |  | 0.112 |
| HPV vaccination |  |  |  |
| Never | / |  | 1.00 |
| Ever | / |  | 0.70(0.10-5.14) |
| Missing | / |  |  |
| P value | / |  | 0.724 |
| Education |  |  |  |
| Illiterate or elementary school | 1.00 |  | 1.00 |
| Junior or senior high school | 0.74(0.43-1.27) |  | 0.90(0.59-1.40) |
| College or above | 0.81(0.40-1.66) |  | 1.19(0.66-2.15) |
| missing |  |  |  |
| P value | 0.537 |  | 0.617 |
| Physical activity at work |  |  |  |
| Light | 1.00 |  | 1.00 |
| Moderate | 0.85(0.42-1.72) |  | 0.62(0.28-1.34) |
| Heavy | 0.90(0.50-1.62) |  | 0.75(0.30-1.88) |
| P value | 0.874 |  | 0.404 |
